# Is gaitrite system sensitive in discriminating gait pattern of subjects affected by Charcot Marie tooth? A pilot study

**DOI:** 10.1007/s10072-025-08535-7

**Published:** 2025-10-18

**Authors:** Cristina Schenone, Maria Lagostina, Marta Ponzano, Chiara Avanti, Cecilia Contenti, Mehrnaz Hamedani, Marina Grandis, Chiara Gemelli, Sara Massucco, Lucio Marinelli, Edoardo Roveta, Angelo Schenone, Carlo Trompetto, Mori Laura

**Affiliations:** 1 Department of Neuroscience, Rehabilitation, Ophthalmology, Genetics, Maternal and Child Health (DINOGMI), University of Genoa, Genoa, Italy; 2 IRCCS Ospedale Policlinico San Martino IRCCS, Genoa, Italy; 3 Department of Health Sciences (DISSAL), Biostatistics Unit, University of Genoa, Genoa, Italy; 4 SC Recupero e Rieducazione Funzionale ASL 3 Genovese, Genoa, Italy

**Keywords:** Hereditary neuropathy, Gait, Balance, Outcome measures

## Abstract

**Background:**

Charcot-Marie-Tooth (CMT) disease is the most common hereditary neuropathy, characterized by progressive distal muscle weakness and gait abnormalities, impacting patients mobility and quality of life. Despite efforts to develop effective treatments, pharmacological options remain limited. Evaluation of gait function is crucial for assessing disease progression and treatment efficacy.

**Aim:**

This study compared the sensitivity of clinical and instrumental outcome measures (OM) in discriminating CMT patients and identifying key spatio-temporal gait parameters and their correlations with clinical measures.

**Methods:**

Eighteen CMT patients and 18 healthy age-matched subjects (HS) were evaluated using clinical scales and instrumental gait analysis. Clinical measures included the 10 m walk test (10MWT), 6-minutes walk test (6MWT), Berg balance scale (BBS), Short Physical Performance Battery (SPPB), CMT Examination Score (CMTES), and Walk12 scale. Instrumental evaluation utilized the GAITRite electronic walkway system to assess spatio-temporal parameters. Correlations between clinical and instrumental parameters were examined.

**Results:**

CMT patients exhibited significantly lower performance in clinical scales compared to HS. Instrumental evaluation revealed significant differences in stride length, velocity, stance percentage, and swing percentage between CMT patients and HS. Correlation analysis demonstrated associations between clinical and instrumental measures, particularly with stride length, gait speed, and balance assessments.

**Conclusion:**

The GAITRite system demonstrated sensitivity in discriminating CMT patients and controls, highlighting gait abnormalities consistent with previous literature findings. Correlations between instrumental and clinical measures suggest potential for objective gait assessment in CMT management. Further research with larger cohorts is warranted to validate these findings and assess longitudinal gait changes in CMT.

## Introduction

Charcot-Marie-Tooth (CMT) disease is the most common hereditary neuropathy with a prevalence of 18 case in 100,000 [1, 2]. Symptoms usually start in the 1st-2nd decade of life with progressive distal muscle weakness and atrophy [3].

There is an important variability in the clinical expression of the disease. However, the involvement of gait is common: ankle and toes dorsiflexors are the most affected muscles and patients often complain of gait disorders with frequent falls and difficulties in running. Additionally, joint tightness, deformities, and altered proprioception further impair gait and balance [4, 5]. Gait abnormalities have long term implications for CMT patients since abnormal loading patterns result in joint and muscle pain [6]. Patients often need aids or orthotics devices, while complete loss of walking ability is rare [3].

There are still no pharmacologic approaches, specific for CMT, although lately the progresses in gene therapy led to major advances in neuromuscular disorders treatment. There are many ongoing trials, and others in preparation, based on experimental evidence in animal models [7]. Rehabilitation and orthotics remain the only possible clinical approach, although efficacy is still unclear [8, 9].

Measuring clinical effects of physiotherapy and other non-pharmacological interventions is essential in research and clinical practice. A common problem is ensuring the repeatability and accuracy of gait evaluation in CMT patients, requiring outcome measures (OM) that can detect changes over time, despite the disease’s slow progression.

The gold standard to assess the disability level is the CMT neuropathy score (CMTNS), a 36-point scale based on symptoms, signs, and neurophysiological measures [10]. To minimize floor and ceiling effects and enhance sensitivity, CMTNS has been updated to a second version (CMTNS2) [11]. Nevertheless, CMTNS2 still has limited capacity to detect the gradual changes, naturally occurring over the disease course [12].

Given the prevalence of ambulation impairment, assessing its characteristics in the CMT patients becomes of clinical importance.

Until now, gait ability in CMT has been commonly quantified by clinical OM such as the 10 m walk test (10MWT) [13, 14] and the 6-minutes walk test (6MWT) [13, 15, 16]. However, clinical tests are not always enough sensitive to detect the impairment and the modifications over time. Another method for assessing gait ability is instrumental gait analysis, which utilizes technological devices to swiftly and accurately measure kinematic parameters in a non-invasive manner. This approach has been suggested as an effective tool for offering a quantitative and comprehensive characterization of walking impairments across various neurological conditions [17–19].

The first aim of the present paper is to confirm the sensitivity of clinical and instrumental OM for gait and balance in discriminating CMT patients. The second aim is to verify the most important spatio-temporal parameters in CMT assessment and their possible correlations with clinical OM.

## Materials and methods

We recruited a group of patients attending the CMT Clinic of IRCCS Ospedale Policlinico San Martino. The study received the approval of the Ethical Committee (N. CET - Liguria: 418/2022 - DB id 11756).

Inclusion criteria: clinical and genetically confirmed diagnosis of CMT; age between 18 and 85 years; ability to walk without support with or without ankle foot orthoses; Short Physical Performance Battery (SPPB) scoring between 2 and 10. Exclusion criteria: other forms of hereditary neuropathy than CMT; vestibular affections; psychiatric, cardiovascular and lung disorders or severe arthropathic changes in the lower limbs; other associated causes of neuropathy.

We evaluated 18 subjects affected by CMT meeting the inclusion criteria. After recording a detailed medical history, complete neurological and physical examinations were performed. All subjects were assessed using clinical scales and gait analysis instruments. We included a control group of 18 healthy subjects, matched by age (HS).

### Clinical evaluation and OM

Walking performances have been investigated with 10MWT and 6MWT. The 10MWT is a validated test that assesses functional mobility and walking speed in m/s over a short distance [13–15]; the 6MWT, already validated in CMT patients, evaluates ambulation ability and aerobic resistance, time, distance and velocity of gait [13, 15, 16, 20].

Balance performances have been evaluated with the Berg Balance Scale (BBS) and SPPB.

BBS, a 14-item objective test, is a sensitive scale to detect subtle balance impairment and fall risk that has been validated in people affected by neurological diseases and has also been used in disability assessment in patients affected by CMT [21, 22]. The total score ranges from 0 to 56, where a score below 45 is indicative of imbalance and great risk of falls [23].

SPPB is widely used to quantify balance and gait impairment in neurological disorders and in CMT [15, 22]. It is a composite measure assessing walking speed, standing balance, and sit-to-stand performance, with a high level of validity, reliability and responsiveness [24]. Total score ranges from 0 to 12, a score below 10 is associated with risk of falls [25, 26].

Disease impairment was evaluated with the CMT Examination Score (CMTES). CMTES is a subscore of total CMTNS that includes 7 items based on patients symptoms and examination findings excluding the electrophysiology, with a maximum total score of 28 points (indicating the worst condition) [27]. Additionally, patients performed a subjective evaluation of walking ability using the Walk12 scale [15, 28].

### Instrumental evaluation

We conducted a gait assessment using the GAITRite system, a 7-meter-long electronic walkway that measures temporal and spatial gait parameters. This non-invasive method requires no attachments to the patient.

As the subject ambulates across the walkway, the pressure exerted by the feet onto the walkway activates the sensors. Patients walked on the carpet for one minute at their usual pace (Normal Walk – NW), at a brisk pace without running (Fast Walk – FW), and at their normal pace while saying aloud words starting with a certain letter (Dual Task – DT). A researcher always walked alongside them for safety.

PKMAS, a software integrated with the GAITRite system, provides data about spatial and temporal parameters of the objects in contact with the walkway surface.

Given the literature regarding the most used parameters [29–31], we focused on: Stride Length, Stride Width, Stride time, Velocity, Cadence. We also checked the different percentage of the Gait Cycle Time: Stance, Swing, Single Support, Total Double Support.

### Statistical analysis

Results were reported as mean (sd) or median (IQR) for continuous variables and as absolute number (%) for categorical variables, overall and separately for CMT and HC subjects. The two groups were compared using Chi-squared test or Mann-Whitney test respectively for categorical and continuous variables. Spearman correlation coefficients were calculated to evaluate the variables correlation in the CMT subjects subgroup.

The level of significance was set to 0.05 and all the statistical analyses were performed using Stata version 16.0 (Stata Corporation, College Station, TX, USA).

## Results

The 18 CMT subjects (10 females; 55.5%) had a mean age of 54.8 years old and a range of 27–76 yy, 13 with CMT1A, 2 CMTX1, 2 CMT1B and 1 CMT4C. See Table 1 for demographic and clinical characteristics. The 18 HS in the control group had a mean age of 51.8 years old and a range of 29-81yy. The two groups were age matched (U = 143.5, *p* = 0.624).

**Table 1:** Clinical and demographic characteristics of CMT patients and HS groups

	Overall(*N* = 36)	CMT(*N* = 18)	HS(*N* = 18)	*p*-value
Age, median(IQR)	57.0 (37.0–65.5.0.5)	58.5 (39.0–68.0)	56.0 (36.0–65.0)	0.624
Male Sex, N(%)	16 (44.4%)	8 (44.4%)	8 (44.4%)	1.000
BMI, median(IQR)	24.0 (21.1–26.3)	26.0 (23.6–27.8)	21.8 (20.1–24.8)	**0.013**
CMT type, N(%)				
CMT1A	---	13(72.2%)	---	---
CMT1B	---	2(11.1%)	---	---
CMT1X	---	2(11.1%)	---	---
CMT4C	---	1(5.6%)	---	---

### Clinical OM

**Fig. 1:**
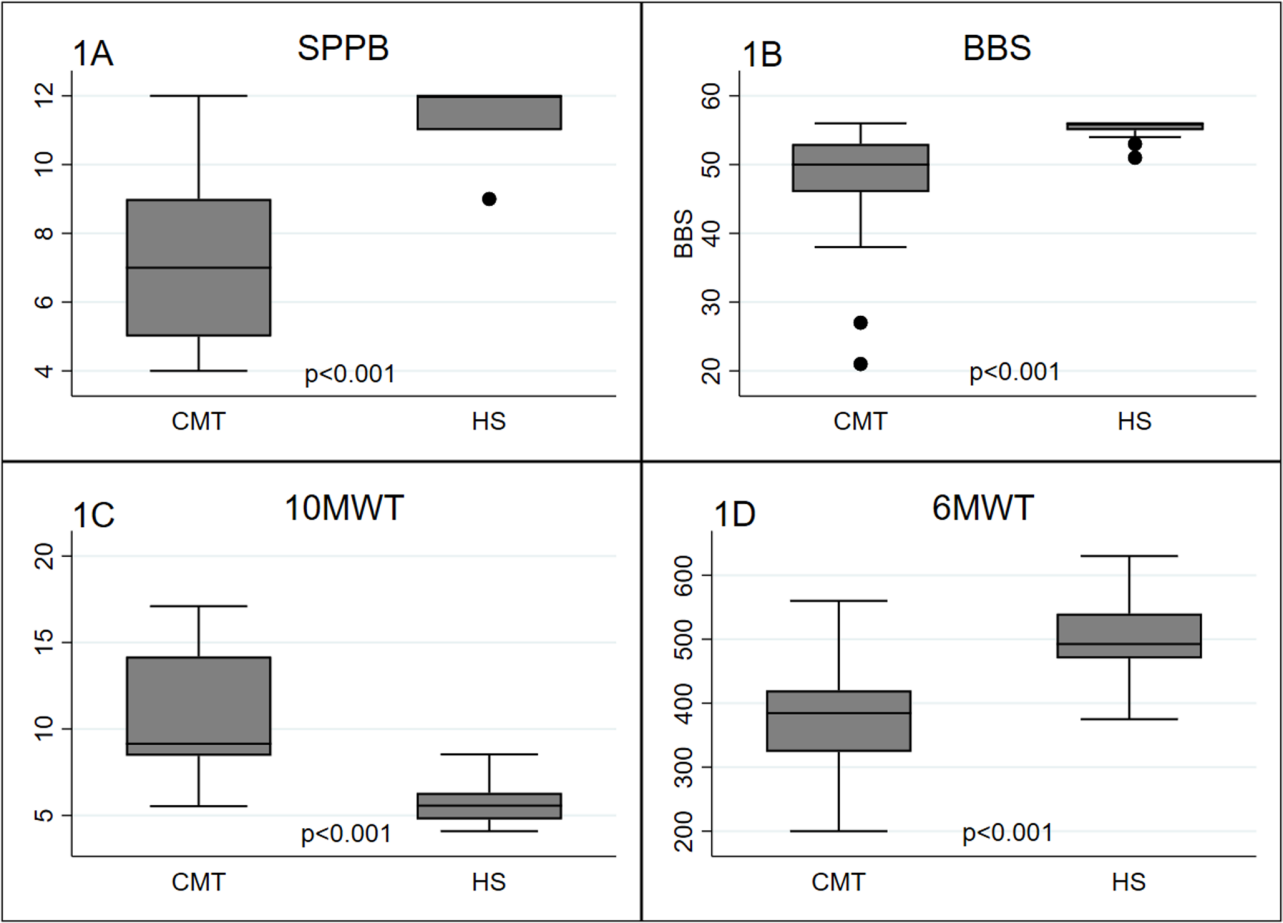
Comparison of CMT and HS performances at the clinical outcome measures. 1 A Short Physical Performance Battery (SPPB); 1B Berg Balance Scale; 1 C 10 m Walk Test (10MWT); 1D 6 min Walk Test (6MWT). * *P* < 0.001 indicates significant differences between CMT subjects and HS

For clinical OM see Fig. 1; Table 2.

**Table 2:** Clinical outcome measures scores. * *P* < 0.05 indicates significant differences between CMT subjects and HS. BBS: Berg balance Scale; SPPB: short physical performance Battery; 10MWT: 10 m walking Test; 6MWT: 6 min walking Test; CMTES: CMT examination Score; WALK-12: questionnaire WALK-12;

	Overall(*N* = 36)	CMT(*N* = 18)	HS(*N* = 18)	*p*-value
BBS, median(IQR)	54.5 (50.0–56.0)	50.0 (46.0–53.0)	56.0 (55.0–56.0)	**< 0.001**
SPPB, median(IQR)	11.0 (7.0–12.0)	7.0 (5.0–9.0)	12.0 (11.0–12.0)	**< 0.001**
10MWT, median(IQR)	7.5 (5.5–9.1)	9.1 (8.5–14.2)	5.6 (4.8–6.3)	**< 0.001**
6MWT, median(IQR)	430.0 (380.0–511.5.0.5)	384.5 (324.0–420.0.0.0)	492.5 (470.0–540.0.0.0)	**< 0.001**
CMTES, median(IQR)	---	8.5 (6.0–10.0)	---	---
WALK-12, median(IQR)	---	36.0 (27.0–40.0)	---	---

Concerning clinical measures, we observed a median of 36.00 at the WALK-12 (IQR = 27–40) and a median of 8.50 at the CMTES (IQR = 6–10). Concerning the balance assessment, at the BBS we found a median score of 50 while at the SPPB 7.0. At the walking tests, they spent a median time of 9.14 s performing the 10MWT and they paced a median of 384.5 m at the 6MWT. CMT and HS subjects were significantly different in terms of BBS, SPPB, 6MWT and 10MWT (p-value < 0.001).

CMT subjects performances were significantly lower than HS performances (*p* < 0.001).

### Instrumental evaluation

For instrumental gait parameters see Table 3.

**Table 3:** Spatio-temporal parameters extracted from instrumental gait assessment by means of the gaitrite during walking tasks. * *P* < 0.05 indicates significant differences between CMT subjects and HS. FW: fast walk; NW: normal Walk; DT: dual Task. Stride length: distance from the heel of one foot to the following heel of the same foot (cm). Stride width: distance between a line connecting the two ipsilateral foot heel contacts (the Stride) and the contralateral foot heel contact between those events, measured perpendicular to the Stride (cm). Stride time and gait cycle time: period from first contact of one foot to the following first contact of the same foot (sec). stance time: period when the foot is in contact with the ground (sec). stance percentage: stance time as a percentage of the gait cycle time. swing time: period when the foot is not in contact with the ground (sec). swing percentage: swing time as a percentage of the gait cycle time. single support time: period when only the current foot is in contact with the ground (sec). single support percentage: single support time as a percentage of gait cycle time. total double support time: sum of all periods when both feet are in contact with the ground during stance phase (sec). total double support percentage: total double support time as a percentage of the gait cycle time. Velocity: obtained after dividing the sum of all Stride Length, by the sum of all Stride time (cm/sec). Cadence: number of footfalls minus one, divided by the ambulation time (steps/min)

	Overall(*N* = 36)	CMT(*N* = 18)	HS(*N* = 18)	*p*-value
NW_Stride Length, median(IQR)	123.6 (105.9–135.9.9.9)	111.1 (96.5–123.6.5.6)	134.3 (123.6–141.0)	**< 0.001**
NW_Stride Width, median(IQR)	8.5 (5.5–10.3)	9.0 (5.6–10.5)	8.3 (5.4–9.8)	0.849
NW_Stance%, median(IQR)	65.2 (63.0–66.5.0.5)	65.7 (64.5–68.6)	64.91 (62.11–65.61)	0.054
NW_Swing%, median(IQR)	34.8 (33.5–37.0)	34.3 (31.4–35.5)	35.1 (34.4–37.9)	0.054
NW_Single Support%, median(IQR)	34.2 (31.1–35.8)	34.1 (31.4–35.3)	34.4 (30.8–36.8)	0.752
NW_Total D. Supp%, median(IQR)	31.4 (28.3–36.8)	31.7 (29.5–37.1)	30.7 (26.2–33.4)	0.311
NW_Velocity, median(IQR)	105.5 (92.4–122.4.4.4)	96.4 (79.5–108.1.5.1)	116.4 (102.5–126.5.5.5)	**0.019**
NW_Cadence, median(IQR)	105.1 (95.8–112.9.8.9)	104.4 (95.2–110.8.2.8)	105.68 (96.4–119.4.4.4)	0.467
FW_Stride Length, median(IQR)	136.9 (119.8–145.6.8.6)	125.96 (102.4–139.0)	142.0 (136.3–154.2.3.2)	**0.002**
FW_Stride Width, median(IQR)	8.0 (6.7–9.5)	8.6 (5.6–10.6)	7.8 (7.0–8.6.0.6)	0.448
FW_Stance%, median(IQR)	64.1 (61.5–66.0)	64.5 (63.0–66.3.0.3)	62.1 (53.7–64.9)	**0.046**
FW_Swing%, median(IQR)	35.9 (34.0–38.5.0.5)	35.5 (33.7–37.0)	37.9 (35.1–46.3)	**0.046**
FW_Single Support%, median(IQR)	35.3 (32.6–36.6)	35.7 (33.7–36.9)	34.5 (31.8–36.4)	0.418
FW_Total D. Supp%, median(IQR)	27.8 (24.3–32.6)	28.8 (26.1–32.7)	26.4 (24.3–30.9)	0.301
FW_Velocity, median(IQR)	130.5 (102.5–150.8.5.8)	118.0 (105.3–142.3.3.3)	142.59 (99.8–160.2.8.2)	0.224
FW_Cadence, median(IQR)	118.8 (110.3–126.2.3.2)	117.5 (113.5–126.2.5.2)	120.9 (92.7–127.3.7.3)	0.800
DT_Stride Length, median(IQR)	119.2 (102.7–131.6.7.6)	102.7 (93.7–126.3.7.3)	127.5 (118.5–139.1.5.1)	**0.001**
DT_Stride Width, median(IQR)	8.6 (5.8–11.1)	9.1 (5.2–11.1)	8.4 (6.7–10.2)	0.718
DT_Stance%, median(IQR)	65.1 (63.6–67.3)	66.1 (64.6–68.7)	63.8 (61.4–66.3)	**0.011**
DT_Swing%, median(IQR)	34.9 (32.7–36.4)	34.0 (31.3–35.4)	36.2 (33.7–38.7)	**0.011**
DT_Single Support%, median(IQR)	34.1 (32.0–35.9.0.9)	34.2 (31.5–36.0)	33.9 (32.5–35.8)	0.618
DT_Total D. Supp%, median(IQR)	31.9 (28.0–35.7.0.7)	31.9 (28.4–36.7)	31.0 (27.6–34.9)	0.333
DT_Velocity, median(IQR)	99.1 (77.3–111.8.3.8)	91.9 (71.4–102.7.4.7)	105.9 (80.7–120.1.7.1)	0.098
DT_Cadence, median(IQR)	100.0 (83.0–109.2.0.2)	102.9 (89.8–108.8.8.8)	96.3 (82.4–109.6.4.6)	0.570
Words, median(IQR)	14.0 (9.5–18.5)	11.0(8.0; 15.0)	15.5 (11.0–19.0)	0.087

Comparing the GAITRite data of CMT and HS, at NW we found significant differences in velocity (*p* < 0.05) and Stride Length (*p* < 0.001) and a trend to significance in stance% and swing% (*p* = 0.054); at FW and DT we found significant differences in stride length, stance% and swing% (*p* < 0.05).

### Correlations

**Fig. 2:**
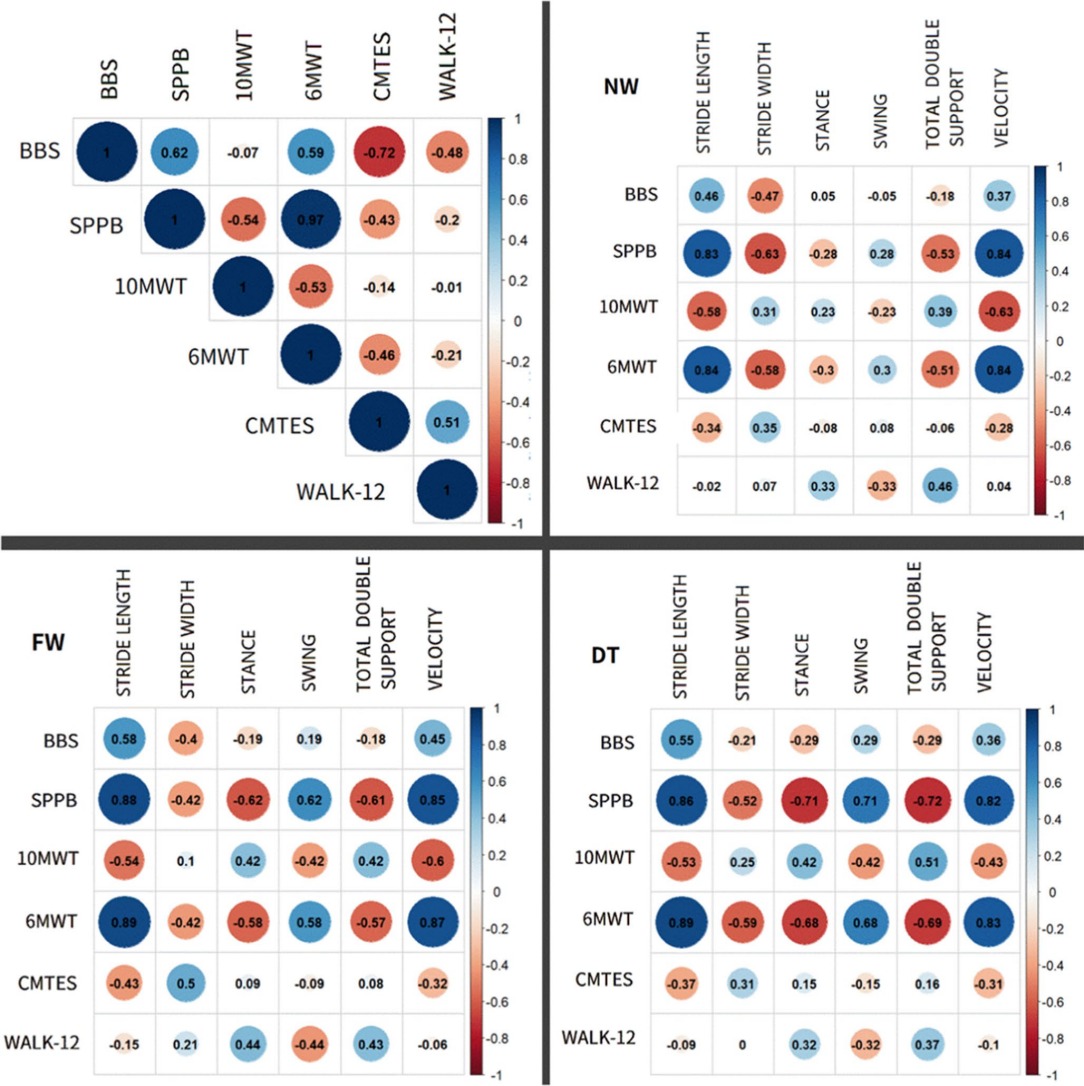
Spearman’s correlation coefficients between clinical outcome measures (OM) and instrumental parameters extracted from instrumental gait assessment by means of the GAITRite in the whole CMT group. The colored circles represent the correlation coefficients, within the range from − 1 (blue) to 1 (red) as indicated by color map at the bottom right. Significant correlations are indicated with *P < 0.05 (Spearman’s correlation test). Upper left panel: correlation between clinical OM; upper right panel: correlation between clinical OM and instrumental parameters during normal walk; lower left panel: correlation between clinical OM and instrumental parameters during fast walk; lower right panel: correlation between clinical OM and instrumental parameters during dual task.BBS: Berg Balance Scale; SPPB: Short Physical Performance Battery; 10MWT: 10 m Walking Test; 6MWT: 6 min Walking Test; CMTES: CMT Examination Score; WALK-12: questionnaire WALK-12; FW: Fast walk; NW: Normal Walk; DT: Dual Task

For clinical OM and instrumental parameters correlations see Fig. 2.

Concerning balance OM, BBS and SPPB present moderate positive correlation (rho 0.62 *p* = 0.006). Concerning walking OM, 10MWT and 6MWT present moderate positive correlation (rho 0.53 *p* = 0.024).

SPPB present a very high positive correlation with 6MWT (rho 0.97 *p* < 0.001), moderate negative correlation with 10MWT (rho − 0.54 *p* = 0.02), and low negative correlation with CMTES, although not significant.

BBS present moderate correlation with 6MWT (rho 0.59 *p* = 0.01), high negative with CMTES (rho − 0.72 *p* < 0.001) and low negative correlation with Walk-12 (rho − 0.48 *p* = 0.043).

6MWT present low negative correlation with CMTES (rho − 0.46 *p* = 0.053). Walk-12 present moderate correlation with CMTES (rho 0.51 *p* = 0.03).

### NW

Stride length present high positive correlation with SPPB (rho 0.83 *p* < 0.001) and 6MWT (rho 0.84 *p* < 0.001), moderate negative correlation with 10 MWT (rho − 0.58 *p* = 0.01).

Stride width present low negative correlation with BBS (rho − 0.47 *p* = 0.047), moderate negative correlation with SPPB (rho − 0.63 *p* = 0.005) and 6MWT (rho − 0.58 *p* = 0.01).

Speed present high positive correlation with SPPB (rho 0.84 pp < 0.001) and 6MWT (rho 0.84 *p* < 0.001) and moderate negative correlation with 10MWT (rho − 0.63 *p* = 0.005).

Total double support% present moderate negative correlation with SPPB (rho − 0.53 *p* = 0.02) and 6MWT (rho − 0.51 *p* = 0.03) and positive correlation with Walk-12 (rho 0.46 *p* = 0.056) although not significant.

### FW

Stride length present significant high positive correlation with SPPB (rho 0.8(*p* < 0.001) and 6MWT (rho 0.89 *p* < 0.001), moderate positive correlation with BBS (rho 0.58 *p* = 0.012) and negative with 10MWT (rho − 0.54 *p* = 0.02).

Stride width present only moderate positive significant correlation with CMTES (rho 0.5 *p* = 0.035).

Speed present significant high positive correlation with SPPB (rho 0.85 *p* < 0.001) and 6MWT (rho 0.87 *p* < 0.001), moderate negative correlation with 10MWT (rho − 0.6 *p* = 0.009).

Stance% present significant moderate negative correlation with SPPB (rho 0.62 *p* = 0.006) and 6MWT (rho − 0.58 *p* = 0.012). Swing% present significant moderate positive correlation with SPPB (rho 0.62 *p* = 0.006) and 6MWT (rho 0.58 *p* = 0.012). Total double support% present significant moderate negative correlation with SPPB (rho − 0.61 *p* = 0.007) and 6MWT (rho − 0.57 *p* = 0.013).

### DT

Stride length present significant high correlation with SPPB (rho 0.86 *p* < 0.001) and 6MWT (rho 0.89 *p* < 0.001), moderate positive correlation with BBS (rho 0.55 *p* = 0.018) and negative with 10MWT (rho − 0.53 *p* = 0.025).

Stride width present significant moderate negative correlation with SPPB (rho − 0.52 *p* = 0.026) and 6MWT (rho − 0.59 *p* = 0.009).

## Discussion and conclusions

The ability to walk independently without tripping and falling is a significant determinant of the quality of life for individuals with CMT [32]. Patients with CMT have an increased risk of non-osteoporotic fractures, primarily occurring in ankle, hand, or foot [33]. Additionally, the risk of falls may be increased by symptoms and signs such as hand cramps, muscle cramps, difficult walking, and weakness. Therefore, it is crucial to assess the risk of falling to implement orthoses and/or aids, thereby mitigating the risk of fractures and complications due to immobilization. In this scenario, the availability of objective OM of gait function sensitive enough to capture changes in the clinical picture without ceiling or floor effects is crucial. Understanding current therapeutic treatment needs, disease progression, patients’ perception of the disease course, and demonstrating the effects of tested interventions reflecting changes in everyday function is one of the research main topics. In fact, recently, there has been considerable international effort to address this, leading to the conclusion that OM should be carefully chosen r to prove the efficacy of a treatment. A poorly designed study using insensitive OM will fail to demonstrate the effectiveness of a treatment or may lead to improperly positive results. In rehabilitation, increasing importance is being placed on instrumental systems that can capture patient performance quickly and objectively. The GAITRite system seems to be sensitive in discriminating between healthy and pathological subjects and in rapidly assessing walking performances during outpatient visits [34–36], even in CMT. As previously mentioned, we chose to investigate the most used spatio-temporal parameters, and we found that in CMT subjects, the most significant parameter is stride length. This data aligns with literature: stride length in our sample is significantly reduced in all tasks compared to healthy subjects, which may be explained by the presence of drop foot or push-off deficits often seen in CMT patients [19]. Multiple factors may contribute to shorter steps. In literature, several studies on children have examined this phenomenon. Particularly, some authors found that the subgroup with the most severe impairment in foot drop and push-off deficit walked significantly slower [18, 37].

The fact that in this pilot study CMT subjects, despite having a mild to moderate impairment at the CMTES, exhibit significantly worse performances compared to the healthy control group, is not surprising, as gait and balance disturbances are among the earliest to manifest and characterize most subtypes of CMT. The median score of 50 on the BBS, indicating a low risk of falls, and the score of 7 on the SPPB, suggesting a moderate risk of falls, may indicate that SPPB scale is more sensitive to capture imbalance; this impression is reinforced by the evidence of the high significant correlation between SPPB and 6MWT, as the latter has already been recognized as a useful test for assessing the gait of these patients [15, 16].

Furthermore, the fact that the 10MWT, the 6MWT, the BBS, and the SPPB are correlated with each other confirms what is already known, as these are useful OM in assessing CMT patients [15].

In our sample, velocity is reduced in CMT subjects only in NW. Gait speed has been suggested as a predictor of functional change in children with CMT [38]; as previously mentioned, speed may be influenced by reduced step length, foot drop during the swing phase with reduced calf power at push-off, and reduced cadence. The non-significance of the data in the other tasks could be related to the small sample size.

The width of the support base does not appear to be increased in our population of individuals with CMT, which is unexpected, as disto-proximal impairment in motor and sensory functions could lead to a wider base of support to prevent imbalance and falls [30]. However, it should be noted that a greater variability of step-to-step base of support in children with CMT has been described compared to the control group [39].

Regarding the percentage of gait phases, we observed a difference only in stance and swing phases in both FW and DT, with a tendency toward significance in NW. We may suggest that since CMT patients have strength deficits and foot deformities with reduced ankle-foot range of motion, these characteristics are more prominently reflected in the stance and swing phases than in other gait cycle phases. Indeed, the imbalance and gait disorder may manifest as shorter steps with reduced swing phase and prolonged stance. The fact that these parameters show only a trend toward significance in NW may be attributed to the small number of recruited subjects.

We also analyzed the possible correlations between clinical and instrumental OM, finding that the SPPB and the 6MWT present the most relevant correlations with all spatio-temporal parameters. This confirms that these scales are capable of discriminating CMT subjects at risk for falls [14, 15] and may be used in current clinical practice. They particularly correlate with stride length, gait speed, and stride width. As for the percentage of gait phases, we observed a correlation only in stance and swing phases in FW and DT, with a trend toward significance in NW.

In conclusion, even with the limited number of participants with various CMT subtypes, we propose that GAITRite could be an effective tool for assessing the walking patterns of CMT patients, as it appears to distinguish CMT subjects from healthy controls. Additionally, the differences detected with GAITRite are consistent with those reported in literature describing the gait features of patients affected by CMT [18, 37, 40], such as stride length and gait speed.

This could also be valuable in CMT longitudinal assessments, aligning with established OM. Nonetheless, these correlations need validation with a larger sample. Future studies should include longer periods for gait assessment. Understanding gait disorders better in CMT patients could lead to tailor-made rehabilitation protocols, enhancing healthcare and quality of life.

## References

[1] Barreto LCLS, Oliveira FS, Nunes PS et al (2016) Epidemiologic study of Charcot-Marie-Tooth disease: a systematic review. Neuroepidemiology 46:157–165. 10.1159/000443706 26849231 10.1159/000443706

[2] Ma M, Li Y, Dai S et al (2023) A meta-analysis on the prevalence of Charcot–Marie–Tooth disease and related inherited peripheral neuropathies. J Neurol 270:2468–2482. 10.1007/s00415-023-11559-8 36631678 10.1007/s00415-023-11559-8

[3] Pareyson D, Marchesi C (2009) Diagnosis, natural history, and management of Charcot–Marie–Tooth disease. Lancet Neurol 8:654–667. 10.1016/S1474-4422(09)70110-3 19539237 10.1016/S1474-4422(09)70110-3

[4] Casasnovas C, Cano LM, Albertí A et al (2008) Charcot-Marie-Tooth disease. Foot Ankle Spec 1:350–354. 10.1177/1938640008326247 19825739 10.1177/1938640008326247

[5] Boffeli TJ, Tabatt JA (2015) Minimally invasive early operative treatment of progressive foot and ankle deformity associated with Charcot-Marie-Tooth disease. J Foot Ankle Surg 54:701–708. 10.1053/j.jfas.2014.03.019 25131389 10.1053/j.jfas.2014.03.019

[6] Nonnekes J, Hofstad C, De Rotteveel A et al (2021) Management of gait impairments in people with hereditary motor and sensory neuropathy: A treatment algorithm. J Rehabil Med 0. 10.2340/16501977-2831 10.2340/16501977-2831 PMC8814859 33880570

[7] Pisciotta C, Pareyson D (2023) Gene therapy and other novel treatment approaches for Charcot-Marie-Tooth disease. Neuromuscul Disord 33:627–635. 10.1016/j.nmd.2023.07.001 37455204 10.1016/j.nmd.2023.07.001

[8] Beloribi-Djefaflia S, Attarian S (2023) Treatment of Charcot-Marie-Tooth neuropathies. Rev Neurol 179:35–48. 10.1016/j.neurol.2022.11.006 36588067 10.1016/j.neurol.2022.11.006

[9] Bertini A, Manganelli F, Fabrizi GM et al (2023) Use, tolerability, benefits and side effects of orthotic devices in Charcot-Marie-Tooth disease. 10.1136/jnnp-2023-332422. J Neurol Neurosurg Psychiatry jnnp-2023-332422 10.1136/jnnp-2023-332422 PMC11041609 37918904

[10] Shy ME, Blake J, Krajewski K et al (2005) Reliability and validity of the CMT neuropathy score as a measure of disability. Neurology 64:1209–1214. 10.1212/01.WNL.0000156517.00615.A3 15824348 10.1212/01.WNL.0000156517.00615.A3

[11] Murphy SM, Herrmann DN, McDermott MP (2011) Reliability of the CMT neuropathy score (second version) in Charcot-Marie‐Tooth disease. Journal of the Peripheral Nervous System 16:191–198. 10.1111/j.1529-8027.2011.00350.x 22003934 10.1111/j.1529-8027.2011.00350.x PMC3754828

[12] Coghe G, Pau M, Mamusa E et al (2020) Quantifying gait impairment in individuals affected by Charcot-Marie-Tooth disease: the usefulness of gait profile score and gait variable score. Disabil Rehabil 42:737–742. 10.1080/09638288.2018.1506946 30334469 10.1080/09638288.2018.1506946

[13] Mannil M, Solari A, Leha A et al (2014) Selected items from the Charcot-Marie-Tooth (CMT) neuropathy score and secondary clinical outcome measures serve as sensitive clinical markers of disease severity in CMT1A patients. Neuromuscul Disord 24:1003–1017. 10.1016/j.nmd.2014.06.431 25085517 10.1016/j.nmd.2014.06.431

[14] Baptista CRD, Nascimento-Elias AH, Garcia B et al (2021) Physical function and performance measures of children and adolescents with Charcot-Marie-Tooth disease. Physiother Theory Pract 37:73–80. 10.1080/09593985.2019.1603257 31046526 10.1080/09593985.2019.1603257

[15] TreSPE Study Group, Mori L, Prada V et al (2019) Outcome measures in the clinical evaluation of ambulatory Charcot-Marie-Tooth 1A subjects. Eur J Phys Rehabil Med 55. 10.23736/S1973-9087.18.05111-0 10.23736/S1973-9087.18.05111-0 29898585

[16] Padua L, Pazzaglia C, Pareyson D et al (2016) Novel outcome measures for Charcot – Marie – Tooth disease: validation and reliability of the 6-min walk test and StepWatch^™^ activity monitor and identification of the walking features related to higher quality of life. Eur J Neurol 23:1343–1350. 10.1111/ene.13033 27160471 10.1111/ene.13033

[17] Lencioni T, Piscosquito G, Rabuffetti M (2017) Responsiveness of gait analysis parameters in a cohort of 71 CMT subjects. Neuromuscul Disord 27:1029–1037. 10.1016/j.nmd.2017.07.003 28844614 10.1016/j.nmd.2017.07.003

[18] Õunpuu S, Garibay E, Solomito M et al (2013) A comprehensive evaluation of the variation in ankle function during gait in children and youth with Charcot–Marie–Tooth disease. Gait Posture 38:900–906. 10.1016/j.gaitpost.2013.04.016 23702343 10.1016/j.gaitpost.2013.04.016

[19] Newman CJ, Walsh M, O’Sullivan R et al (2007) The characteristics of gait in Charcot-Marie-Tooth disease types I and II. Gait Posture 26:120–127. 10.1016/j.gaitpost.2006.08.006 17010610 10.1016/j.gaitpost.2006.08.006

[20] Knak K, Andersen L, Witting N, Vissing J (2017) Reliability of the 2- and 6-minute walk tests in neuromuscular diseases. J Rehabil Med 49:362–366. 10.2340/16501977-2222 28352938 10.2340/16501977-2222

[21] Matjacić Z, Zupan A (2006) Effects of dynamic balance training during standing and stepping in patients with hereditary sensory motor neuropathy. Disabil Rehabil 28:1455–1459. 10.1080/09638280600646169 17166808 10.1080/09638280600646169

[22] Mori L, Signori A, Prada V et al (2020) Treadmill training in patients affected by Charcot–Marie–Tooth neuropathy: results of a multicenter, prospective, randomized, single-blind, controlled study. European Journal of Neurology 27:280–287. 10.1111/ene.14074 31444929 10.1111/ene.14074 PMC6973058

[23] Park S-H, Lee Y-S (2017) The diagnostic accuracy of the Berg balance scale in predicting falls. West J Nurs Res 39:1502–1525. 10.1177/0193945916670894 27784833 10.1177/0193945916670894

[24] Treacy D, Hassett L (2018) The short physical performance battery. J Physiother 64:61. 10.1016/j.jphys.2017.04.002 28645532 10.1016/j.jphys.2017.04.002

[25] Lauretani F, Ticinesi A, Gionti L et al (2019) Short-physical performance battery (SPPB) score is associated with falls in older outpatients. Aging Clin Exp Res 31:1435–1442. 10.1007/s40520-018-1082-y 30515724 10.1007/s40520-018-1082-y

[26] Welch SA, Ward RE, Beauchamp MK et al (2021) The short physical performance battery (SPPB): a quick and useful tool for fall risk stratification among older primary care patients. J Am Med Dir Assoc 22:1646–1651. 10.1016/j.jamda.2020.09.038 33191134 10.1016/j.jamda.2020.09.038 PMC8113335

[27] Fridman V, Sillau S, Acsadi G et al (2020) A longitudinal study of CMT1A using Rasch analysis based CMT neuropathy and examination scores. Neurology 94. 10.1212/WNL.0000000000009035 10.1212/WNL.0000000000009035 PMC7238948 32047073

[28] Pagliano E, Moroni I, Baranello G et al (2011) Outcome measures for Charcot-Marie‐Tooth disease: clinical and neurofunctional assessment in children. Journal of the Peripheral Nervous System 16:237–242. 10.1111/j.1529-8027.2011.00357.x 22003938 10.1111/j.1529-8027.2011.00357.x PMC3917107

[29] Bower K, Thilarajah S, Pua Y-H et al (2019) Dynamic balance and instrumented gait variables are independent predictors of falls following stroke. J Neuroeng Rehabil 16:3. 10.1186/s12984-018-0478-4 30612584 10.1186/s12984-018-0478-4 PMC6322221

[30] Kwon M-S, Kwon Y-R, Park Y-S, Kim J-W (2018) Comparison of gait patterns in elderly fallers and non-fallers. Technol Health Care 26:427–436. 10.3233/THC-174736 29758966 10.3233/THC-174736 PMC6004957

[31] Lai Y-R, Lien C-Y, Huang C-C et al (2022) Clinical disease severity mediates the relationship between stride length and speed and the risk of falling in Parkinson’s disease. JPM 12:192. 10.3390/jpm12020192 35207680 10.3390/jpm12020192 PMC8875632

[32] Padua L, Shy ME, Aprile I et al (2008) Correlation between clinical/neurophysiological findings and quality of life in Charcot-Marie-Tooth type 1A. J Peripher Nerv Syst 13:64–70. 10.1111/j.1529-8027.2008.00159.x 18346232 10.1111/j.1529-8027.2008.00159.x

[33] Pouwels S, De Boer A, Leufkens HGM et al (2014) Risk of fracture in patients with Charcot–Marie–Tooth disease. Muscle Nerve 50:919–924. 10.1002/mus.24240 24634316 10.1002/mus.24240

[34] Webster KE, Wittwer JE, Feller JA (2005) Validity of the GAITRite^®^ walkway system for the measurement of averaged and individual step parameters of gait. Gait Posture 22:317–321. 10.1016/j.gaitpost.2004.10.005 16274913 10.1016/j.gaitpost.2004.10.005

[35] Sosnoff JJ, Weikert M, Dlugonski D et al (2011) Quantifying gait impairment in multiple sclerosis using GAITRite^™^ technology. Gait Posture 34:145–147. 10.1016/j.gaitpost.2011.03.020 21531562 10.1016/j.gaitpost.2011.03.020

[36] Schmidheiny A, Swanenburg J, Straumann D et al (2015) Discriminant validity and test re-test reproducibility of a gait assessment in patients with vestibular dysfunction. BMC Ear Nose Throat Disord 15:6. 10.1186/s12901-015-0019-8 26500447 10.1186/s12901-015-0019-8 PMC4619276

[37] Ferrarin M, Bovi G, Rabuffetti M et al (2012) Gait pattern classification in children with Charcot–Marie–Tooth disease type 1A. Gait Posture 35:131–137. 10.1016/j.gaitpost.2011.08.023 21944474 10.1016/j.gaitpost.2011.08.023 PMC3909942

[38] Kennedy R, Carroll K, Paterson KL et al (2017) Deterioration in gait and functional ambulation in children and adolescents with Charcot–Marie–Tooth disease over 12 months. Neuromuscul Disord 27:658–666. 10.1016/j.nmd.2017.04.005 28495045 10.1016/j.nmd.2017.04.005

[39] Kennedy RA, McGinley JL, Paterson KL et al (2018) Gait and footwear in children and adolescents with Charcot-Marie-Tooth disease: a cross-sectional, case-controlled study. Gait Posture 62:262–267. 10.1016/j.gaitpost.2018.03.029 29579702 10.1016/j.gaitpost.2018.03.029

[40] Kennedy RA, Carroll K, McGinley JL (2016) Gait in children and adolescents with Charcot-Marie‐Tooth disease: a systematic review. J Peripher Nerv Syst 21:317–328. 10.1111/jns.12183 27513454 10.1111/jns.12183

